# Lactoferrin restrains allergen-induced pleurisy in mice

**DOI:** 10.1007/s00011-012-0522-y

**Published:** 2012-07-19

**Authors:** Michał Zimecki, Jolanta Artym, Maja Kocięba, Katarzyna Kaleta-Kuratewicz, Marian L. Kruzel

**Affiliations:** 1 Institute of Immunology and Experimental Therapy, Wrocław, Poland; 2 Wrocław University of Environmental and Life Sciences, Wroclaw, Poland; 3 Department of Integrative Biology and Pharmacology, University of Texas Health Science Center at Houston, 6341 Fannin Street, MSB 4.200, Houston, TX 77030 USA

**Keywords:** Allergy, Buccal, Lactoferrin, Mice, Pleurisy

## Abstract

**Objectives:**

The aim of this study was to assess the utility of lactoferrin (LF), a natural immunomodulator, to restrain allergen-induced pleurisy in mice.

**Material and subjects:**

BALB/c female mice, 8- to 10-week old, weighing 24 g on average, were used.

**Treatment:**

Mice were immunized intraperitoneally with 50 μg of ovalbumin (OVA) and the pleurisy was elicited 14 days later by intrapleural injection of 12.5 μg of OVA. LF was given 24 and 3 h before elicitation of the allergic reaction.

**Methods:**

The cytokine levels in the pleural exudates were measured by immunoassays. The blood and pleural exudates smears were stained with Giemsa and May-Grünwald reagents and reviewed histologically. Lung sections were stained with eosin and hematoxylin for histological evaluation.

**Results:**

Lactoferrin significantly decreased manifestation of pleurisy induced by OVA in a sensitized mouse model. In particular, the percentages of eosinophils in blood and pleural exudates were strongly diminished. The histological analysis of lungs revealed that LF diminished the development of pathological lesions, such as pulmonary edema, diffuse alveolar hemorrhage and hemosiderosis, which were found in the lungs after injection of the eliciting dose of OVA. LF also decreased the level of IL-5 secreted into the pleural fluid.

**Conclusions:**

This is a first demonstration that LF significantly decreases antigen-specific pleurisy in a sensitized mouse model.

## Introduction

Allergy constitutes a global and aggravating health problem affecting primarily populations in developed countries. It is estimated that up to 300 million people worldwide experience allergic asthma and these estimates are expected to rise as the incidence of asthma has been steadily increasing. Etiology of the disease comprises both genetic and environmental factors, including infectious agents and air pollutants. Asthma is dependent on the presence of antigen-specific IgE antibodies [1] and Th2 type cells [2, 3]. Airways inflammation is thought to play a central role in the pathogenesis of asthma with mast cells [4] and eosinophils [5] being responsible for molecular responses via secretion of cytokines and low-molecular weight mediators.

The therapeutic approaches in allergy encompass subcutaneous (SCIT) and, more recently, sublingual (SLIT) immunotherapies (reviewed by [6]). In the experimental models in mice a key role in protection against allergens is played by regulatory T cells and TGF-beta [7, 8]. However, it is suggested [9] that immune deviation of allergen-specific Th2 cells may be more effective than induction of general immune response suppression.

Among various agents capable to suppress allergy in an antigen nonspecific manner, are biologically active peptides and proteins of therapeutic value, classified as biopharmaceuticals [10] found, among others, in whey and milk casein [11]. For example, whey protein hydrolysate suppressed atopic dermatitis in mice [12] and formulas containing hydrolyzed casein and whey were found as effective as breast milk to prevent allergy in infants [13].

In this investigation we have used a mouse model of ovalbumin (OVA)-induced allergic pulmonary inflammation which has been well established to induce airways eosinophilia and extensive lung damage analogous to that seen in asthma [14]. It is the first attempt to evaluate the effects of lactoferrin (LF), a natural immunomodulator, on amelioration of OVA-induced lung pathology.

Lactoferrin is an evolutionally old iron-binding protein, contained in body fluids and neutrophils, playing an important role in maintaining immune homeostasis [15, 16]. LF affects both physiological and pathological immunological responses and, in the latter case, abnormal Th1/Th2 balance can be corrected by LF [17]. Although there exists a vast literature regarding successful immune interventions in infectious, inflammatory and autoimmune disorders by LF, very few reports describe beneficial actions of LF in allergy mediated by IgE antibodies and Th2 cells [18, 19]. Recently, LF was shown to inhibit pollen antigen-induced allergic airway inflammation in mice as assessed by histology of airways and reactive oxygen species in culture of bronchial epithelial cells [20]. Interestingly, the robust inflammatory processes were significantly prevented by LF administration but not by a classical iron binding synthetic compound desferroxamine (DFO) [21]. Although both LF and DFO share some characteristics of iron binding, LF is able to diminish these symptoms more efficiently than is DFO. Thus, it is clear that LF has additional activities, that may be due to its affinity to bind LPS, heparin, lysozyme, or DNA [22], which determine its action in OVA-induced pleurisy. In a sheep model it was also shown that LF abolishes both late-phase bronchoconstriction and airway hyperresponsiveness by the means of heparin binding and inactivation of mast cell tryptase [18]. Others demonstrated that LF decreases the release of the allergic mediator histamine when incubated with skin mast cells [19].

The aim of this investigation was to evaluate effectiveness of buccal and other ways of administration of LF in prevention of OVA-induced pleurisy in mice.

## Materials and methods

### Mice

BALB/c female mice, 8 to 10 weeks old, were delivered by the Institute of Laboratory Medicine, Łódź, Poland. The mice were fed a commercial, granulated food and water ad libitum. The local ethics committee approved the study.

### Reagents

Bovine LF (BLF) from milk was from Tatua Co-operative Dairy Company, New Zealand (<15 % iron saturation, <10 endotoxin U/mg) and human milk LF (HLF) from Sigma (cat. no L0520). Both LFs were low endotoxin and iron saturation less than 15 %. Dexamethasone (Dexaven) was from Jelfa, Poland, Maalox (aluminum hydroxide 3.5 g and magnesium hydroxide 4.0 g in 100 ml) from Rhone-Poulenc Rorer, France, Narcotan^®^ from Leciva, Czech Republik. ELISA kit for determination of IL-5 was from Becton Dickinson (cat. no 555236) and ELISA kit for IFN-γ determination was from eBioscience (cat. no 88-7314-88). Endotoxin free OVA, trypan blue, Giemsa, May-Gruünwald, haematoxylin, eosin, toluidine blue and formalin were from Sigma-Aldrich Corporation, St. Louis, MO, USA.

### Sensitization of mice with OVA

Mice were immunized intraperitoneally (i.p.) with 50 μg of OVA in 0.2 ml of Maalox (adjuvant) as described elsewhere [23]. After 14 days mice were given the eliciting dose of OVA intrapleurally (12.5 μg in 50 μl of 0.9 % NaCl). This group of mice is later referred to as sensitized control mice. Mice treated i.p. only with Maalox but received OVA, intrapleurally, are referred to in the text and figure legends as the background (BG) group.

### The mode of lactoferrin administration

Lactoferrin (50–800 μg) was given buccally in 25 μl of 0.9 % NaCl/dose, 24 and 3 h before administration of the eliciting dose of OVA. Alternatively, LF was administered by gavage intragastrically (500 μg/dose in 0.2 ml of 0.9 % NaCl) or by an intraperitoneal injection (500 μg/dose in 0.2 ml of 0.9 % NaCl). These mice are referred as LF groups and labeled in the figures and tables as BLF or HLF.

### In vivo and ex vivo protocols

Mice were subjected to halothane anesthesia for blood collection by the retro-orbital plexus. Mice were killed by cervical dislocation and organs were collected as described below.

### Assessment of the blood cell composition

Blood smears were prepared on microscope glasses, dried and stained with Giemsa and May-Grünwald reagents. The smears were subsequently reviewed under the light microscope at 1,000× magnification. The blood cell compositions were presented as a percentage of a given cell type: neutrophil precursors (band forms), mature neutrophils (segments), eosinophils, lymphocytes and monocytes.

### Determination of the pleural exudates cell number, composition and cytokine level

The pleural exudates and lungs were collected from mice after the pleural cavities were washed with 0.2 ml of 0.9 % NaCl containing EDTA (10 mM) for each cavity. Fifty (50 μl) of the pleural lavage was taken for determination of cell number, the lavage then was centrifuged and the supernatant was saved for cytokine determination (frozen at −20 °C). From the cell pellet a smear was prepared, dried and stained with Giemsa and May-Grünwald reagents. Cell numbers were enumerated in a Bürker hemocytometer. The cell types composition in the pleural fluid was determined by an independent histologist at 1,000× magnification. The exudates cell compositions were presented as a percentage of a given cell type: neutrophil precursors (band forms), mature neutrophils (segments), eosinophils, basophils, lymphocytes, monocytes, macrophages and mastocytes.

The concentrations of IL-5 and IFN-γ in the pleural fluid were measured by ELISA kits according to manufacturer’s instructions.

### Histological analysis

The lungs removed from each mouse were fixed in 4 % formalin solution for 48 h, washed, dehydrated, cleared in xylene and embedded in paraffin. The paraffin embedded tissues were sectioned in a Micron HM310 microtome into 6 μm sections. The sections were stained with hematoxylin and eosin (H&E) and viewed under Nikon Eclipse 801 microscope. The pathologist viewing and interpreting all histological sections was blinded to the type of experiment and treatment.

### Statistics

The results of one representative experiment from three independent experiments were presented. The results are presented as mean values ± standard error (SE). Brown–Forsyth’s test was used to determine the homogeneity of variance between the groups. When the variance was homogenous, analysis of variance (one-way ANOVA) was applied, followed by post hoc comparisons with the Tukey’s test to estimate the significance of the differences between groups. Nonparametric data were evaluated with the Kruskal–Wallis’ ANOVA, as indicated in the text. Significance was determined at *P* < 0.05. Statistical analysis was performed using STATISTICA 7 for Windows.

## Results

### Inhibition of OVA-induced pleurisy by lactoferrin: effects on cell numbers and cell composition in the circulating blood and pleural exudates

In a pilot experiment we showed that BLF (500 μg/dose), given intraperitoneally or intragastrically, significantly attenuated symptoms of OVA-induced allergy as evaluated by reduced cell numbers in pleural exudates and diminished content of eosinophils in the circulating blood (data not shown).

**Fig. 1:**
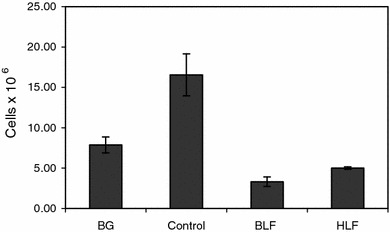
The cell number in the pleural exudates. Mice were sensitized with OVA and the allergic reaction to OVA was elicited after 14 days as described in “Materials and methods”. Human or bovine LF was administered buccally (500 μg dose) at 24 and 3 h before the eliciting dose of antigen. The cell numbers in the pleural exudates were determined 24 h later. The results are presented as the mean values from five mice/group ± SE. Statistics: BG versus control, *P* = 0.0027; control versus BLF, *P* = 0.0002; control versus HLF, *P* = 0.0003 (ANOVA)

**Fig. 2:**
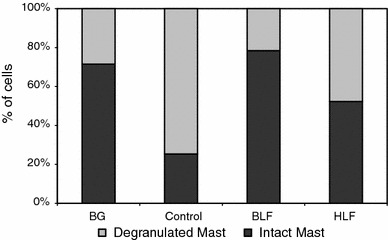
The percentage of intact and de-granulated mastocytes in the pleural exudates. The experiment was performed as described in Fig. 1. 24 h after OVA challenge the percentages of intact and de-granulated mastocytes were determined. Statistics: de-granulated and intact mastocytes: BG versus control, *P* = 0.0001; control versus BLF, *P* = 0.0001; control versus HLF, *P* = 0.0015 (ANOVA). *NS* not significant

In a second experiment the effects of BLF and HLF, given buccally, were studied to assess the suppression of OVA-induced pleurisy. The results showed that although both LFs deeply suppressed the numbers of cells in the exudates (below BG levels), the action of bovine LF was stronger (Fig. 1). Likewise, BLF was slightly more effective in normalization of blood cell composition (Table 1) and number of de-granulated mastocytes in the pleural exudates (Fig. 2).

**Table 1:** The cell composition in the circulating blood

Experimental group	Cell types in the circulating blood				
	B	S	Eo	L	Mono
BG	1.60 ± 0.51	19.80 ± 2.48	3.00 ± 0.00	75.60 ± 3.08	0.00 ± 0.00
Control	5.40 ± 0.93	28.50 ± 0.92	9.00 ± 0.00	55.40 ± 1.91	0.40 ± 0.24
BLF	2.80 ± 0.49	28.75 ± 2.42	4.40 ± 0.40	66.33 ± 0.48	0.00 ± 0.00
HLF	4.00 ± 0.00	28.20 ± 1.74	5.00 ± 0.00	63.40 ± 2.42	0.00 ± 0.00

The experiment was performed as described in Fig. 1. 24 h after elicitation of the allergic reaction the blood pictures were analyzed. The content of respective cell types in the blood is given in percentage (mean values from five mice) ± SE

Statistics: B (Bands): BG versus control, *P* = 0.0016; control versus BLF, *P* = 0.0284; control versus HLF NS (*P* = 0.3568) (ANOVA); S (Segments): BG versus control, *P* = 0.0322; control versus BLF NS (*P* = 0.9997); control versus HLF NS (*P* = 0.9995) (ANOVA); Eo (Eosinophils): BG versus control *P* = 0.0001; control versus BLF, *P* = 0.0001; control versus HLF, *P* = 0.0001 (ANOVA); L (Lymphocytes): BG versus control, *P* = 0.0002; control versus BLF, *P* = 0.0134; control versus HLF NS (*P* = 0.1885) (ANOVA); Mono (Monocytes): BG versus control NS (*P* = 0.1374); control versus BLF NS (*P* = 0.1374); control versus HLF NS (*P* = 0.1374) (ANOVA)

*NS* not significant

**Fig. 3:**
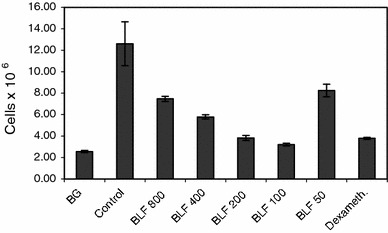
The cell number in the pleural exudates. Mice were immunized with OVA and the allergic pleurisy reaction was elicited after 14 days as described in “Materials and methods”. The mice were treated buccally with 800–50 μg doses of BLF at 24 and 3 h before the eliciting dose of antigen. Dexamethasone was used at a single dose of 20 μg, intraperitoneally, 3 h before elicitation of the allergic response. 24 h after elicitation of the response the numbers of cells in the pleural exudates were determined. The results were presented as the mean values from five mice/group ± SE. Statistics: BG versus control, *P* = 0.0001; control versus BLF 800, *P* = 0.0011; control versus BLF 400, *P* = 0.0001; control versus BLF 200, *P* = 0.0001; control versus BLF 100, *P* = 0.0001; control versus BLF 50, *P* = 0.0074; control versus dexameth., *P* = 0.0001 (ANOVA)

Another experiment was aimed at establishing the optimal dose of LF using buccal administration of the protein. BLF was given at a dose range of 800–50 μg/mouse buccally and dexamethasone, a reference anti-inflammatory drug, at a single dose of 20 μg intraperitoneally, at 24 and 3 h before challenge with eliciting dose of OVA. Figure 3 shows a significant (almost a sixfold) increase of the cell numbers in the pleural exudates of the sensitized control mice over BG levels. The treatment of mice with decreasing doses of LF revealed an increasing, protective, anti-inflammatory effect with the optimal effect at 100 μg dose. The dose of 50 μg was much less effective. Therefore, in subsequent experiments the 100 μg dose of LF was used. The effect of dexamethasone was comparable to that of 100 μg of LF.

The protective effect of LF was also evident when analyzing the blood cell composition (Table 2). Sensitized mice displayed a higher content of neutrophils and, in particular, eosinophils (a fourfold increase). The treatment of mice with LF decreased the percentage of eosinophils with a best effect at 100 μg dose. On the other hand, the percentage of neutrophil precursors (band forms) was elevated. The application of dexamethasone evoked similar changes.

**Table 2:** The cell composition of the circulating blood

Experimental group	Cell types in the circulating blood				
	B	S	Eo	L	Mono
BG	1.20 ± 0.37	23.75 ± 1.24	2.75 ± 0.19	72.25 ± 1.36	0.00 ± 0.00
Control	3.00 ± 0.00	31.60 ± 2.42	11.20 ± 0.86	54.75 ± 0.86	0.80 ± 0.37
BLF 800	7.40 ± 0.75	31.75 ± 0.49	5.60 ± 0.51	55.40 ± 1.40	0.40 ± 0.24
BLF 400	5.80 ± 0.37	27.20 ± 1.88	6.40 ± 0.60	60.20 ± 2.06	0.40 ± 0.24
BLF 200	6.00 ± 0.45	29.60 ± 1.72	3.60 ± 0.51	56.50 ± 0.74	2.40 ± 0.68
BLF 100	6.40 ± 0.60	26.25 ± 0.92	3.60 ± 0.40	59.60 ± 2.01	3.00 ± 0.45
BLF 50	6.00 ± 0.32	35.25 ± 1.28	9.00 ± 1.05	52.20 ± 2.85	0.40 ± 0.24
Dexameth.	4.00 ± 0.71	30.40 ± 1.89	5.00 ± 0.00	59.00 ± 2.97	1.00 ± 0.00

The experiment was performed as described in Fig. 3. 24 h after elicitation of the allergic reaction the blood pictures of mice were analyzed. The contents of respective cell types in the blood is given in percentage (mean values from 5 mice) ± SE

Statistics: B (Bands): BG versus control NS (*P* = 0.2134); control versus BLF 800, *P* = 0.0001; control versus BLF 400, *P* = 0.0083; control versus BLF 200, *P* = 0.0039; control versus BLF 100, *P* = 0.0009; control versus BLF 50, *P* = 0.0039; control versus dexameth. NS (*P* = 0.8441) (ANOVA); S (Segments): BG versus control, *P* = 0.0271; control vs BLF 800 NS (*P* = 1.0000); control versus BLF 400 NS (*P* = 0.5230); control versus BLF 200 NS (*P* = 0.9848); control versus BLF 100 NS (*P* = 0.2838); control versus BLF 50 NS (*P* = 0.7320); control versus dexameth. NS (*P* = 0.9993) (ANOVA); Eo (Eosinophils): BG versus control, *P* = 0.0001; control versus BLF 800, *P* = 0.0002; control versus BLF 400, *P* = 0.0002; control versus BLF 200, *P* = 0.0001; control versus BLF 100, *P* = 0.0001; control versus BLF 50 NS (*P* = 0.2183); control versus dexameth., *P* = 0.0001 (ANOVA); L (Lymphocytes): BG versus control, *P* = 0.0001; control versus BLF 800 NS (*P* = 0.9999); control versus BLF 400 NS (*P* = 0.5105); control versus BLF 200 NS (*P* = 0.9980); control versus BLF 100 NS (*P* = 0.6489); control versus BLF 50 NS (*P* = 0.9811); control versus dexameth. NS (*P* = 0.7785) (ANOVA); Mono (Monocytes): BG versus control NS (*P* = 1.0000); control versus BLF 800 NS (*P* = 1.0000); control versus BLF 400 NS (*P* = 1.0000); control versus BLF 200 NS (*P* = 0.7695); control versus BLF 100 NS (*P* = 1.0000); control versus BLF 50 NS (*P* = 1.0000); control versus dexameth. NS (*P* = 1.0000) (ANOVA of Kruskal–Wallis)

*NS* not significant

The analysis of cell composition in the pleural exudates (Table 3) led to a similar conclusion as in the case of blood cell composition. In sensitized mice there was a 3.3-fold increase of eosinophils. The treatment of mice with LF resulted in a decrease in number of eosinophil content, lowest observed at 200–100 μg doses. At 800 and 400 μg doses the appearance of neutrophil precursors was also noted. In addition, the treatment of mice with LF or dexamethasone induced a higher percentage of macrophages in the exudates.

**Table 3:** The cell composition of the pleural exudates

Experimental group	Cell types in the pleural exudates							
	B	S	Eo	Baso	L	Mono	Mø	Mast
BG	0.00 ± 0.00	17.80 ± 1.28	7.60 ± 0.40	1.40 ± 0.75	40.80 ± 1.66	4.75 ± 0.37	22.60 ± 1.03	6.00 ± 0.00
Control	0.00 ± 0.00	16.00 ± 0.00	25.20 ± 0.73	4.00 ± 0.00	19.00 ± 1.58	1.60 ± 0.81	11.80 ± 1.20	22.20 ± 1.32
BLF 800	3.40 ± 0.93	23.80 ± 0.86	9.25 ± 0.37	0.00 ± 0.00	24.75 ± 0.37	3.50 ± 0.22	18.00 ± 0.32	14.80 ± 0.73
BLF 400	3.00 ± 0.45	17.00 ± 1.10	7.80 ± 0.58	0.00 ± 0.00	24.00 ± 1.67	4.40 ± 0.24	24.75 ± 0.37	20.00 ± 1.30
BLF 200	0.00 ± 0.00	12.40 ± 1.57	6.25 ± 0.19	0.00 ± 0.00	27.66 ± 0.18	3.60 ± 1.03	22.60 ± 0.87	26.20 ± 2.11
BLF 100	0.00 ± 0.00	21.40 ± 0.93	6.50 ± 0.22	0.00 ± 0.00	23.00 ± 1.90	4.00 ± 0.00	23.80 ± 1.36	21.00 ± 2.24
BLF 50	0.00 ± 0.00	20.80 ± 1.36	20.40 ± 1.25	0.00 ± 0.00	16.60 ± 1.63	0.00 ± 0.00	11.50 ± 0.67	28.66 ± 0.37
Dexameth.	0.00 ± 0.00	25.00 ± 2.88	8.00 ± 1.70	0.00 ± 0.00	15.40 ± 3.85	0.00 ± 0.00	27.60 ± 2.23	24.00 ± 2.88

The experiment was performed as described in Fig. 3. 24 h after elicitation of the allergic response the cell compositions in the pleural exudates were analyzed. The content of respective cell types is given in percentage (mean values from 5 mice) ± SE

Statistics: B (Bands): BG versus control NS (*P* = 1.0000); control versus BLF 800, *P* = 0.0001; control versus BLF 400, *P* = 0.0001; control versus BLF 200 NS (*P* = 1.0000); control versus BLF 100 NS (*P* = 1.0000); control versus BLF 50 NS (*P* = 1.0000); control versus dexameth. NS (*P* = 1.0000) (ANOVA); S (Segments): BG versus control NS (*P* = 0.9866); control versus BLF 800, *P* = 0.0133; control versus BLF 400 NS (*P* = 0.9996); control versus BLF 200 NS (*P* = 0.6597); control versus BLF 100 NS (*P* = 0.1872); control versus BLF 50 NS (*P* = 0.3115); control versus dexameth., *P* = 0.0029 (ANOVA); Eo (Eosinophils): BG versus control, *P* = 0.0001; control versus BLF 800, *P* = 0.0001; control versus BLF 400, P = 0.0001; control versus BLF 200, *P* = 0.0001; control versus BLF 100, *P* = 0.0001; control versus BLF 50, *P* = 0.0073; control versus dexameth., *P* = 0.0001 (ANOVA); Baso (Basophils): all comparisons NS (*P* = 0.1262) (ANOVA of Kruskal–Wallis); L (Lymphocytes): BG versus control, *P* = 0.0001; control versus BLF 800 NS (*P* = 0.4224); control versus BLF 400 NS (*P* = 0.5950); control versus BLF 200 NS (*P* = 0.0549); control versus BLF 100 NS (*P* = 0.8135); control versus BLF 50 NS (*P* = 0.9853); control versus dexameth. NS (*P* = 0.8807) (ANOVA); Mono (Monocytes): BG versus control, *P* = 0.0020; control versus BLF 800 NS (*P* = 0.1566); control versus BLF 400, *P* = 0.0077; control versus BLF 200 NS (*P* = 0.1175); control versus BLF 100, *P* = 0.0325; control versus BLF 50 NS (*P* = 0.3342); control versus dexameth. NS (*P* = 0.3342) (ANOVA); Mø (Macrophages): BG versus control, *P* = 0.0001; control versus BLF 800, *P* = 0.0132; control versus BLF 400, *P* = 0.0001; control versus BLF 200, *P* = 0.0001; control versus BLF 100, *P* = 0.0001; control versus BLF 50 NS (*P* = 1.0000); control versus dexameth., *P* = 0.0001 (ANOVA); Mast (Mastocytes): BG versus control, *P* = 0.0001; control versus BLF 800 NS (*P* = 0.0592); control versus BLF 400 NS (*P* = 0.9793); control versus BLF 200 NS (*P* = 0.6802); control versus BLF 100 NS (*P* = 0.9995); control versus BLF 50 NS (*P* = 0.1400); control versus dexameth. NS (*P* = 0.9936) (ANOVA)

*NS* not significant

**Fig. 4:**
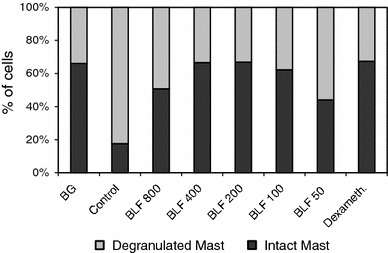
The percentages of intact and de-granulated mastocytes in the pleural exudates. The experiment was performed as described in Fig. 3. 24 h after elicitation of the allergic response the percentages of intact and de-granulated mastocytes in the pleural exudates were determined. Statistics: de-granulated and intact mastocytes: BG versus control, *P* = 0.0001; control versus BLF 800, *P* = 0.0001; control versus BLF 400, *P* = 0.0001; control versus BLF 200, *P* = 0.0001; control versus BLF 100, *P* = 0.0001; control versus BLF 50, *P* = 0.0001; control versus dexameth., *P* = 0.0001 (ANOVA)

Significant differences were also observed in the proportions of intact and de-granulated mastocytes (Fig. 4). In control, sensitized mice, the percentage of de-granulated mastocytes was exceptionally high (82.4 %). LF or dexamethasone decreased the percentage of de-granulated mastocytes to the BG levels.

### Effect of lactoferrin on cytokine production

**Fig. 5:**
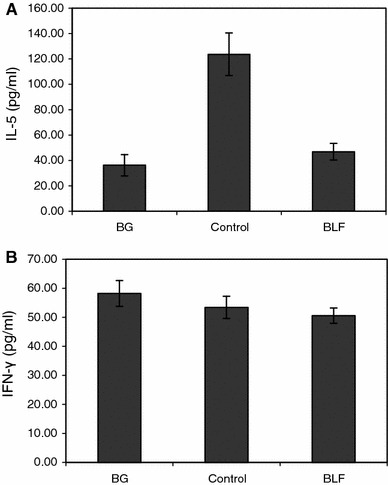
IL-5 (**a**) and IFN-γ (**b**) concentration in the pleural exudates. Mice were sensitized with OVA and the allergic pleurisy reaction was elicited after 14 days as described in “Materials and methods”. BLF (100 μg/dose) was given buccally to mice 24 and 3 h before OVA challenge. The pleural exudates were harvested 24 h later and the cytokine concentrations were determined by ELISA. The results were shown in picograms/ml of IL-5 or IFN-γ as mean values from 5 mice ± SE. Statistics: **a** BG versus control, *P* = 0.0001; control versus BLF, *P* = 0.0003 (ANOVA); **b** BG versus control NS (*P* = 0.6345); control versus BLF NS (*P* = 0.8510) (ANOVA). *NS* not significant

Cytokines, in particular IL-5, are involved in mediation of the allergic inflammation. We found that IL-5 concentration, measured in the pleural fluid, was significantly elevated in sensitized mice as compared to the appropriate BG group (Fig. 5a). The pretreatment of mice with BLF (100 μg/mouse, buccally) reversed that phenomenon. On the other hand, IFN-γ levels were similar in sensitized and BG mice (Fig. 5b).

### Lactoferrin decreases pathological changes in lungs of sensitized mice

**Fig. 6:**
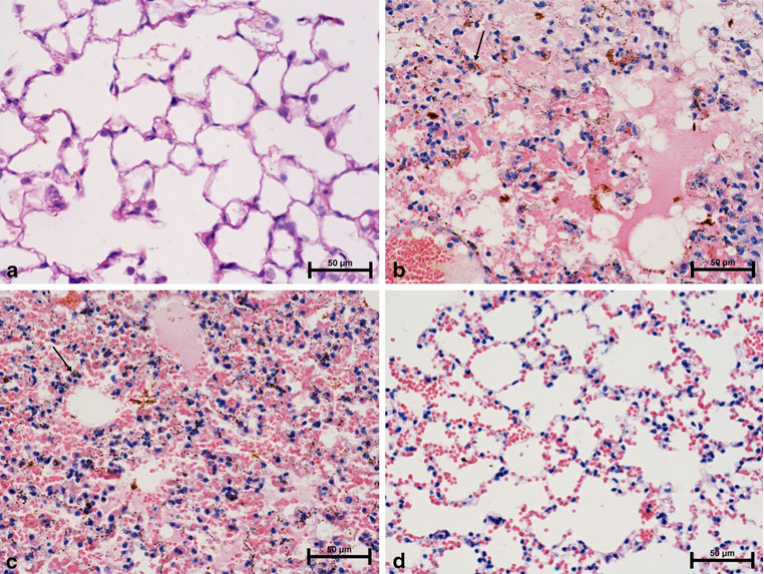
Effect of LF on OVA-induced lung injury—histopathological changes. Mice were immunized with OVA and the allergic pleurisy reaction was elicited after 14 days as described in “Materials and methods”. Bovine LF (100 μg/dose) was given bucally to mice 24 and 3 h before elicitation of the allergic response. The histological analysis of lungs was performed 24 h later. **a** naive mice; **b**, **c** control, sensitized mice (OVA-sensitized mice which were given the eliciting dose of OVA and no LF); **d** mice treated with of BLF. **a** The lung of naive mouse (normal appearance); **b** severe edema and hemosiderin deposits in the lungs of sensitized control mouse; **c** alveolar hemorrhage and hemosiderosis in the lung of sensitized control mouse; **d** lung congestion of LF-treated mouse. *Arrows* indicate siderophages, H&E, original magnification× 400

Histological analysis of OVA-sensitized mice revealed severe pulmonary edema (Fig. 6b). Edematous fluid was detected in the alveolar septa (interstitial pulmonary edema), within alveoli (alveolar pulmonary edema) and also within bronchioles. Edematous lesions were especially advanced in the subpleural tissue. Extensive interstitial and alveolar hemorrhages (Fig. 6c) were also observed. In the zones of extravasations the phenomenon of hemosiderosis was detected. That phenomenon consisted of appearance of hemosiderin-laden macrophages (siderophages) and hemosiderin deposits in the alveolar spaces and within alveolar septa. Moderate inflammatory infiltrations, formed by mononuclear cells around the bronchioles, blood vessels and in the interstitium, were also present.

Treatment of mice with BLF (100 μg, buccally, 24 and 3 h) before OVA challenge restrained the development of pathological changes in the lungs. There were no signs of edema, hemorrhages and hemosiderosis in the lungs. The histological examination demonstrated an early stage of lung congestion (Fig. 6d). The lung capillaries were all uniformly widened and filled with erythrocytes. Alveolar septa were atonic. Focal infiltrations of interstitium and subpleural zone were noted. In addition, mild inflammatory infiltrates were located around bronchioles and blood vessels.

## Discussion

The results presented in this report demonstrated effectiveness of LF in lessening of OVA-induced pleurisy, a well-established experimental mouse model of asthma. These findings are in accordance with a generally accepted view on the immune modulatory action of LF [17]. Our previous studies revealed that oral administration of LF to volunteers [24] regulated cytokine production by blood cell cultures and elicited neutrophil release into circulation. On the other hand, oral application of LF before surgery attenuated postsurgical decline in immune reactivity [25]. In mice, LF given in drinking water, reversed suppression of the immune response caused by the immobilization stress [26]. Also LF has been shown to protect against immune-mediated tissue damage in various experimental models. For example, mice treated with BLF had increased survival and decreased gut tissue destruction after LPS injection [27]. This also holds true for damage elicited by mycobacterial antigen trehalose 6,6″-dimycolate [28]. Additionally, LF added together with BCG vaccine resulted in increased protection against an aerosol TB challenge, with evidence of decreased lung damage [29]. Orally administered LF proved also to be protective in viral [30] and fungal [31] infections in mice.

Although the effectiveness of oral administration of LF is well established, the mechanism(s) are still under investigation. We postulate that LF may interact with cells present in the lamina propria of the oral cavity such as dendritic cells, macrophages and T lymphocytes via LF receptors (e.g. TLR, CD14, mannose receptor or sialoadhesin). Resident lingual CD4^+^ T lymphocytes, bearing TLR2 and TLR4 and comprising both suppressive T cells and cells with effector function seem to be of a primary importance [32]. These cells are able to produce main regulatory cytokines (IFN-γ, IL-4, IL-10 and IL-17) upon antigenic stimulation. It is, therefore, conceivable that these cells may proliferate and penetrate the circulation and the lymphatic system thus altering the immune reactivity upon second antigenic challenge.

Here we report significant decrease in numbers of eosinophils in the blood and pleural exudates following application of LF. That phenomenon was well correlated with the decrease of IL-5 concentration in the pleural fluid (Fig. 5a) and is in accord with generally accepted views on the interrelationship between eosinophil number and IL-5. IL-5 is a major maturation and differentiation factor for eosinophils [33] and belongs also to Th2-type cytokines involved in development of allergic immune responses [34]. Taking into account that LF had no effect on IFN-γ level (Fig. 5b) one can assume that the shift to Th1-type response was the major mechanism of the protective LF action in this model.

However, considering various modes of anti-inflammatory actions of LF, other phenomena may also contribute to the overall inhibitory effect of LF in OVA-induced pleurisy. For example, although administration of LF did not lower the mastocytes content in the pleural exudates (Table 3) it suppressed mastocyte de-granulation (Figs. 2, 4). That could be a consequence of inactivation of proteolytic mast cell enzymes by LF [18, 19]. In addition, the increase in macrophage and neutrophil contents in the pleural exudates (Table 3) may also play a role in inhibition of Th2-mediated allergy [35, 36] by mechanisms involving increased IFN-γ production.

The histological analysis revealed only an early phase of lung congestion in the LF-treated mice. Such lesions precede development of alveolar hemorrhages and pulmonary edema, which were observed in the lung of sensitized mice. On the basis of the histological examination we concluded that LF prevented the progress of the above mentioned lesions.

Of interest, the effective dose range of LF was rather narrow, i.e. 800 μg/dose was too high and 50 μg not enough to exert a strong suppressive effect. Moreover, at 800 and 400 μg doses the appearance of neutrophil precursors was noted, probably associated with the ability of LF to induce myelopoiesis at high doses [37]. In addition, the suppression of the allergic reaction was comparable for bovine and human milk LFs. Lastly, buccal, intragastric and intraperitoneal routes of LF administrations were similarly effective suggesting involvement of the same receptor responsible for the anti-inflammatory effect. LF as an evolutionary old protein interacts with receptors associated with innate immunity, widely distributed throughout the body [38]. Toll like receptors, in particular TLR4, are good candidates for mediation of LF protective action in this experimental model since this protein interacts with TLR4 via the carbohydrate chains [39]. Interestingly, ligation of TLR4 on oral Langerhans cells is important to develop tolerogenic state to pathogens [40]. Among TLRs (TLR1-8) the expression of TLR4 is significantly lower on peripheral blood cells of asthmatic patients [41] and it was also associated with a decreased ex vivo production of Th1-type and anti-inflammatory cytokines by these cells. It is, therefore, conceivable that activation of cells by LF via TLR4 may lead to suppression of the allergic immune response described in this work. Additional studies will be necessary to determine LF’s role in maintaining immune homeostasis during allergic insults. Such studies should be grounded in the precept that the impact of LF on the regulation of physiological parameters is conditioned by the physiological state of the host.

In summary, this investigation expanded our knowledge on the suppressive effect of LF in allergy and suggests that buccal application of LF may be effective in amelioration of allergy symptoms in patients.

## References

[1] Yssel H, Abbal C, Pene J, Bousquet J (1998). The role of IgE in asthma. Clin Exp Allergy.

[2] Del Prete G (1992). Human Th1 and Th2 lymphocytes: their role in the pathology of atopy. Allergy.

[3] Corry DB, Kheradmand F (2009). Toward a comprehensive understanding of allergic lung disease. Trans Am Clin Climatol Assoc.

[4] Bradding P, Walls AF, Holgate ST (2006). The role of the mast cell in the pathophysiology of asthma. J Allergy Clin Immunol.

[5] Rådinger M, Lötvall J (2009). Eosinophil progenitors in allergy and asthma: do they matter?. Pharmacol Ther.

[6] Incorvaia C, Frati F (2011). One century of allergen-specific immunotherapy for respiratory allergy. Immunotherapy.

[7] Chung Y, Lee SH, Kim DH, Kang CY (2005). Complementary role of CD4+CD25+ regulatory T cells and TGF-beta in oral tolerance. J Leukoc Biol.

[8] Strickland DH, Stumbles PA, Zosky GR, Subrata LS, Thomas JA, Turner DJ, Sly PD, Holt PG (2006). Reversal of airway hyperresponsiveness by induction of airway mucosal CD4+CD25+ regulatory T cells. J Exp Med.

[9] Romagnani S (2006). Regulatory T cells: which role in the pathogenesis and treatment of allergic disorders?. Allergy.

[10] Antosova Z, Mackova M, Kral V, Macek T (2009). Therapeutic application of peptides and proteins: parenteral forever?. Trends Biotechnol.

[11] Zimecki M, Kruzel ML (2007). Milk-derived proteins and peptides of potential therapeutic and nutritive value. J Exp Ther Oncol.

[12] Shimizu N, Dairiki K, Ogawa S, Kaneko T (2006). Dietary whey protein hydrolysate suppresses development of atopic dermatitis-like skin lesions induced by mite antigen in NC/Nga mice. Allergol Int.

[13] Hays T, Wood RA (2005). A systemic review of the role of hydrolyzed infant formulas in allergy prevention. Arch Pediatr Adolesc Med.

[14] Kumar RK, Herbert C, Foster PS (2008). The ‘classical’ ovalbumin challenge model of asthma in mice. Curr Drug Targets.

[15] Baveye S, Elass E, Mazurier J, Spik G, Legrand D (1999). Lactoferrin: a multifunctional glycoprotein involved in the modulation of the inflammatory process. Clin Chem Lab Med.

[16] Legrand D, Mazurier J (2010). A critical review of the roles of host lactoferrin in immunity. Biometals.

[17] Fischer R, Debbabi H, Dubarry M, Boyaka P, Tome D (2006). Regulation of physiological and pathological Th1 and Th2 responses by lactoferrin. Biochem Cell Biol.

[18] Elrod KC, Moore WR, Abraham WM, Tanaka RD (1997). Lactoferrin, a potent tryptase inhibitor, abolishes late-phase airway responses in allergic sheep. Am J Respir Crit Care Med.

[19] He S, McEuen AR, Blewett SA, Li P, Buckley MG, Leufkens P, Walls AF (2003). The inhibition of mast cell activation by neutrophil lactoferrin: uptake by mast cells and interaction with tryptase, chymase and catepsin G. Biochem Pharmacol.

[20] Kruzel ML, Bacsi A, Choudhury B, Sur S, Boldogh I (2006). Lactoferrin decreases pollen antigen-induced allergic airway inflammation in a murine model of asthma. Immunology.

[21] Chodaczek G, Saavedra-Molina A, Bacsi A, Kruzel ML, Sur S, Boldogh I (2007). Iron-mediated dismutation of superoxide anion augments antigen-induced allergic inflammation: effect of lactoferrin. Postepy Hig Med Dosw (online).

[22] van Berkel PH, Geerts ME, van Veen HA, Mericskay M, de Boer HA, Nuijens JH (1997). N-terminal stretch Arg2, Arg3, Arg4 and Arg5 of human lactoferrin is essential for binding to heparin, bacterial lipopolysaccharide, human lysozyme and DNA. Biochem J.

[23] Bandeira-Melo C, Bonavita AG, Diaz BL, E Silva PM, Carvalho VF, PJ Jose, Flower RJ, Perretti M, MA Martins (2005). A novel effect for annexin 1-derived peptide ac2-26: reduction of allergic inflammation in the rat. J Pharmacol Exp Ther.

[24] Zimecki M, Właszczyk A, Cheneau P, Brunel AS, Mazurier J, Spik G, Kubler A (1998). Immunoregulatory effects of a nutritional preparation containing bovine lactoferrin taken orally by healthy individuals. Arch Immunol Exp Ther (Warsz).

[25] Zimecki M, Właszczyk A, Wojciechowski R, Dawiskiba J, Kruzel M (2001). Lactoferrin regulates the immune responses in post-surgical patients. Arch Immunol Ther Exp (Warsz).

[26] Zimecki M, Artym J, Chodaczek G, Kocięba M, Kruzel M (2005). Effects of lactoferrin on the immune response modified by the immobilization stress. Pharmacol Rep.

[27] Kruzel ML, Harari Y, Chen CY, Castro GA (2000). Lactoferrin protects gut mucosal integrity during endotoxemia induced by lipopolysaccharide in mice. Inflammation.

[28] Welsh KJ, Hwang SA, Hunter RL, Kruzel ML, Actor JK (2010). Lactoferrin modulation of mycobacterial cord factor trehalose 6-6′-dimycolate induced granulomatous response. Transl Res..

[29] Hwang SA, Wilk K, Kruzel ML, Actor JK (2009). A novel recombinant human lactoferrin augments the BCG vaccine and protects alveolar integrity upon infection with *Mycobacterium tuberculosis* in mice. Vaccine.

[30] Wakabayashi H, Kurokawa M, Shin K, Teraguchi S, Tamura Y, Shiraki K (2004). Oral lactoferrin prevents body weight loss and increases cytokine responses during herpes simplex virus type 1 infection of mice. Biosci Biotechnol Biochem.

[31] Takakura N, Wakabayashi H, Ishibashi H, Yamauchi K, Teraguchi S, Tamura Y, Yamaguchi H, Abe S (2004). Effect of orally administered bovine lactoferrin on the immune response in the oral candidiasis murine model. J Med Microbiol.

[32] Mascarell L, Lombardi V, Zimmer A, Louise A, Tourdot S, Van Overtvelt L, Moingeon P (2009). Mapping of the lingual immune system reveals the presence of both regulatory and effector CD4+ T cells. Clin Exp Allergy.

[33] Kouro T, Takatsu K (2009). IL-5- and eosinophil-mediated inflammation: from discovery to therapy. Int Immunol.

[34] Anderson GP, Coyle AJ (1994). TH2 and ‘TH2-like’ cells in allergy and asthma: pharmacological perspectives. Trends Pharmacol Sci.

[35] Tang C, Inman MD, van Rooijen N, Yang P, Shen H, Matsumoto K, O’Byrne PM (2001). Th type 1-stimulating activity of lung macrophages inhibits Th2-mediated allergic airway inflammation by an IFN-gamma-dependent mechanism. J Immunol.

[36] Nance S, Cross R, Yi AK, Fitzpatrick EA (2005). IFN-gamma production by innate immune cells is sufficient for development of hypersensitivity pneumonitis. Eur J Immunol.

[37] Zimecki M, Artym J, Kocięba M (2009). Endogenous steroids are responsible for lactoferrin-induced myelopoiesis in mice. Pharmacol Rep.

[38] Suzuki YA, Lopez V, Lonnerdal B (2005). Mammalian lactoferrin receptors: structure and function. Cell Mol Life Sci.

[39] Ando K, Hasegawa K, Shindo K, Furusawa T, Fujino T, Kikugawa K, Nakano H, Takeuchi O, Akira S, Akiyama T, Gohda J, Inoue J, Hayakawa M (2010). Human lactoferrin activates NF-kappa B through the Toll-like receptor 4 pathway while it interferes with the lipopolysaccharide-stimulated TLR4 signalling. FEBS J.

[40] Allam JP, Peng WM, Appel T, Wenghoefer M, Niederhagen B, Bieber T, Berge S, Novak N (2008). Toll-like receptor 4 ligation enforces tolerogenic properties of oral mucosal Langerhans cells. J Allergy Clin Immunol.

[41] Lun SW, Wong CK, Ko FW, Hui DS, Lam CW (2009). Expression and functional analysis of toll-like receptors of peripheral blood cells in asthmatic patients: implication for immunopathological mechanism in asthma. J Clin Immunol.

